# Altered T Follicular Helper Cell Subsets and Function in Chronic Lymphocytic Leukemia

**DOI:** 10.3389/fonc.2021.674492

**Published:** 2021-04-28

**Authors:** Xun Wu, J. Ernesto Fajardo-Despaigne, Christine Zhang, Vishala Neppalli, Versha Banerji, James B. Johnston, Spencer B. Gibson, Aaron J. Marshall

**Affiliations:** ^1^ Department of Immunology, Max Rady College of Medicine, Rady Faculty of Health Sciences, University of Manitoba, Winnipeg, MB, Canada; ^2^ Hematopathology Laboratory, Shared Health Manitoba, Winnipeg, MB, Canada; ^3^ Research Institute in Oncology and Hematology, CancerCare Manitoba, Winnipeg, MB, Canada; ^4^ Department of Internal Medicine, Max Rady College of Medicine, Rady Faculty of Health Sciences, University of Manitoba, Winnipeg, MB, Canada; ^5^ Department of Biochemistry and Medical Genetics, Max Rady College of Medicine, Rady Faculty of Health Sciences, University of Manitoba, Winnipeg, MB, Canada

**Keywords:** chronic lymphocytic leukemia (CLL), T follicular helper (Tfh) cell, interleukin 21 (IL-21), ibrutinib, TIGIT, coculture assay, bone marrow

## Abstract

Follicular helper T cells (T_FH_) have specialized properties in promoting normal B cell activation but their role in chronic lymphocytic leukemia (CLL) is unknown. We find that T_FH_ cells are elevated in CLL patients and are phenotypically abnormal, expressing higher levels of PD-1, TIGIT, CD40L, IFNγ and IL-21, and exhibiting abnormal composition of T_FH_1, T_FH_2 and T_FH_17 subsets. Frequencies of CD4-positive T cells expressing T_FH_1 markers and IL-21 were positively correlated with patient lymphocyte counts and RAI stage, suggesting that accumulation of abnormal T_FH_ cells is concomitant with expansion of the leukemic B cell clone. Treatment with ibrutinib led to normalization of T_FH_ frequencies and phenotype. T_FH_ cells identified in CLL bone marrow display elevated expression of several functional markers compared to blood T_FH_ cells. CLL T cell-B cell co-culture experiments revealed a correlation of patient T_FH_ frequencies with functional ability of their CD4-positive T cells to promote CLL proliferation. Conversely, CLL cells can preferentially activate the T_FH_ cell subset in co-culture. Together our results indicate that CLL development is associated with expansion of abnormal T_FH_ populations that produce elevated levels of cytokines and costimulatory molecules which may help support CLL proliferation.

## Highlights

1. Follicular helper T cells with altered cytokine and receptor profiles are progressively expanded in CLL and normalized upon treatment.
2. CLL B cells can preferentially activate follicular helper T cells, promoting CD4^+^ T cell-driven CLL B cell proliferation *in vitro*.

## Introduction

Monoclonal B cell lymphocytosis (MBL) and chronic lymphocytic leukemia (CLL) are lymphoproliferative disorders characterized by the presence of abnormal numbers of CD5+ monoclonal B lymphocytes in the blood or tissues (1). MBL is the precursor to CLL, with approximately 1% of high-count cases requiring therapy each year following progressing to CLL (2). The clinical course of CLL patients is heterogeneous and prognostic markers have been developed to predict which patients may have aggressive disease (3). Independent prognostic markers for CLL include Rai stage, age, *IGVH* mutational status, β2-microglobulin level and TP53 loss-of-function (1, 3), which are used to calculate the International Prognostic Index (4). Within tissue microenvironments, CLL B cells come into close contact with other cells such as stromal cells, which provide signals that promote survival and drug resistance (5). The lymphoid tissue environment also promotes activation of B cell antigen receptor (BCR) signaling pathways and CLL proliferation (6).

Although CLL has historically been characterized as a disease of enhanced cell survival, active signaling and proliferation within lymphoid tissue is now appreciated to be an important factor determining disease prognosis (7, 8). Within bone marrow, spleen and lymph nodes, CLL proliferation occurs in “proliferation centers” where CLL cells interact directly with T cells, myeloid cells and stromal cells and display markers of active signaling (9). CLL patients showing highly active proliferation centers exhibit aggressive disease and poor prognosis (10). Inhibitors of Bruton’s Tyrosine Kinase (BTK) have proven to be efficacious in treating CLL *via* interrupting BCR signaling as well as the supportive cell:cell interactions within the lymphoid tissue microenvironment (11). BTK inhibitor treatment of CLL patients frequently results in a transient increase in circulating malignant cells after treatment, concomitant with dramatic loss of leukemic cells from lymph nodes (12), suggesting that these treatments trigger a rapid dissolution of proliferation centers.

Autologous human T cells were found to be required for CLL proliferation in a mouse xenograft model (13), suggesting they play an essential role distinct from stromal cells. Normal B cell follicles, as well as germinal centers containing activated B cells, are known to depend on a specialized subset of CD4+ T cells called follicular helper T cells (T_FH_). These CD4+ T cells express the chemokine receptor CXCR5 (14, 15), that allows them to migrate toward its ligand CXCL13, the B cell follicle chemokine made by follicular dendritic cells (16, 17). Normal T_FH_ cells produce a unique spectrum of cytokines and costimulatory molecules and provide essential co-stimulatory signals to sustain B cell survival and proliferation within germinal centers (18). Functionally distinct T_FH_ subpopulations have been identified based on their differential expression of CXCR3 and CCR6 (19, 20). Abnormalities in T_FH_ populations have been observed in a number autoimmune diseases, where considerable evidence implicates them as drivers of pathological B cell responses (19). While substantial evidence indicates that T cell populations are altered in CLL (21–23), a full assessment of T_FH_ populations across the spectrum of MBL and CLL has not previously been reported.

Here we report a comprehensive assessment of T_FH_ populations and associations of their frequency and phenotypes with CLL biomarkers, clinical stage and immune dysfunction. We find evidence that CLL T_FH_ exhibit an increased functional capacity to produce co-stimulatory receptors and cytokines linked to CLL survival and proliferation and skewing to a T_FH_1-like phenotype in advanced stage patients. Finally, we find that CLL cells can preferentially activate T_FH_ cells *in vitro* and observe an association of T_FH_ frequencies with the ability of activated CD4+ T cells to trigger CLL proliferation. These results define alterations in T_FH_ phenotype and function in CLL and indicate a potential role for these cells as part of the dysfunctional immune microenvironment in this disease.

## Methods

### Patient Samples and Clinical Biomarkers

Peripheral blood and bone marrow aspirates were obtained from CLL patients attending the CLL clinic at CancerCare Manitoba. Informed consent of patients and control subjects was obtained under a protocol approved the Research Ethics Board at the University of Manitoba. Rai staging was determined using standard clinical criteria. Clinical biomarkers including CD38, IgM, IgG, IgA and lymphocyte count were determined using standard protocols and obtained from the Manitoba Tumor Bank and CAISIS database. Mononuclear cells were isolated using Ficoll-Paque density gradient and cryopreserved in 10% DMSO medium prior to analysis. Lymph node biopsies were formalin-fixed and paraffin-embedded prior to sectioning.

### T Cell Phenotyping

For assessment of T_FH_ subpopulations, peripheral blood or bone marrow mononuclear cells were stained for the markers CD3, CD4, CD14, CD19, CXCR5, PD-1, ICOS, CD45RA, CCR7, CXCR3, CCR6, TIGIT (antibody details in **Supplementary Table 1**) and LIVE/DEAD™ Fixable Aqua viability dye (Invitrogen™) at room temperature for 30 minutes. Stained cells were run on a Beckman Coulter Cytoflex instrument. T_FH_ populations were quantified as percent of the singlet, CD4+, Dump (CD19/CD14/LiveDead) negative population as illustrated in **Supplementary Figure 1**.

### Production of Costimulatory Molecules and Cytokines

For assessment of cytokine production, cryopreserved peripheral blood mononuclear cells (PBMC) were cultured overnight and then stimulated for 6 hours with 50ng/ml PMA and 1μg/ml ionomycin (Selleck Chemicals), with 10μg/ml Brefeldin A (Selleck) added for the last 4 hours. Cells were then cell surface stained for CD3, CD14, CD19, CXCR5, CCR6, CXCR3 as described above, fixed and permeabilized using eBioscience™ Fixation/Permeabilization buffer and stained intracellularly for CD4, IFNγ, IL-21 and CD40L at room temperature for 45 minutes. Intracellular CD4 staining was utilized to improve discrimination of CD4+ cells, as cell surface CD4 is downmodulated upon treatment with PMA+ionomycin. Antibody details are provided in **Supplementary Table 1**.

### Immunofluorescence Microscopy

Lymph node tissue sections were deparaffinized, rehydrated and boiled for 20 minutes in Target Retrieval Solution, Citrate pH 6.1 (Agilent). After washing, serial tissue sections were blocked with 1% BSA and 2% FBS in PBS followed by staining with unconjugated primary antibodies at 4°C overnight. After washing, sections were incubated with secondary antibodies for 4 hours at room temperature with shaking, washed and then stained with directly conjugated primary antibodies at 4°C overnight. Following a final wash, sections were air dried and mounted with ProLong™ Gold antifade reagent (Invitrogen) and kept at -20°C until analysis. Antibody details are provided in **Supplementary Table 2**. Images were captured with a CSU-X1M5000 spinning disc confocal microscope (Carl Zeiss) equipped with 405/488/561/635nm lasers.

### CD4 T Cell: CLL B Cell Co-Culture Assay

Autologous CD4 T cells and CLL B cells were purified from PBMC using EasySep™ Human CD4+ T Cell Isolation Kit and EasySep™ Human B Cell Enrichment Kit II without CD43 Depletion (both STEMCELL Technologies), respectively. Purified CD4 T cells were suspended at 1x10^6 cells/mL in RPMI 1640 media (GE Healthcare) with 10% FBS (Life Technologies). T cells were cultured overnight in 24-well plates with/without addition of ImmunoCult™ Human CD3/CD28/CD2 T Cell Activator (STEMCELL Technologies) at 25 μL cocktail/mL of cells. B cells were cultured overnight in U-bottom 96-well plates at 1x10^6 cells/well, in the presence of sCD40L+IL-4 (both 50ng/mL; R&D Systems). After 14-16 hours incubation, cells were washed to remove stimuli. B cells were stained with carboxyfluorescein succinimidyl ester (CFSE) (Sigma-Aldrich) at 0.3 μM in PBS for 5min at room temperature, then washed with culture media. T cells (with or without pre-activation) were co-cultured together with CFSE-labelled autologous CLL-B cells in U-bottom 96-well plates, using 2x10^5 T and 1x10^6 B cells per well. T and B cells alone were included as controls. Starting at day 2 of co-culture, 100 μL of culture medium was gently taken out and fresh medium added to each well daily. At indicated times, wells were harvested and flow cytometry analyses were carried out using the panel detailed in **Supplementary Table 1**. Briefly, cells were stained for CD4, CD19, CXCR5, CXCR3, CCR6, CCR7, CD69, CD25, CD134/OX40, PD-1, CD38 and LIVE/DEAD™ Fixable Aqua viability dye (Invitrogen™). Following wash, cells were fixed and permeabilized as above and stained for Ki-67 at room temperature for 45 min.

### Data Analysis and Statistics

Flow data were analyzed by FlowJo^®^ V10 (FlowJo, LLC). Statistical analysis was performed with GraphPad Prism (GraphPad Software Inc). Confocal images were processed by ImageJ (V1.47). Box and whisker plots illustrate the median, interquartile range and 10-90% percentile values. Statistical tests used are indicated in figure legends and differences were considered to be statistically significant at values of *(p<0.05), **(p<0.01), ***(p<0.001) and ****(p<0.0001).

## Results

### Follicular Helper T Cells Are Expanded and Phenotypically Distinct in CLL Patients

**Figure 1:**
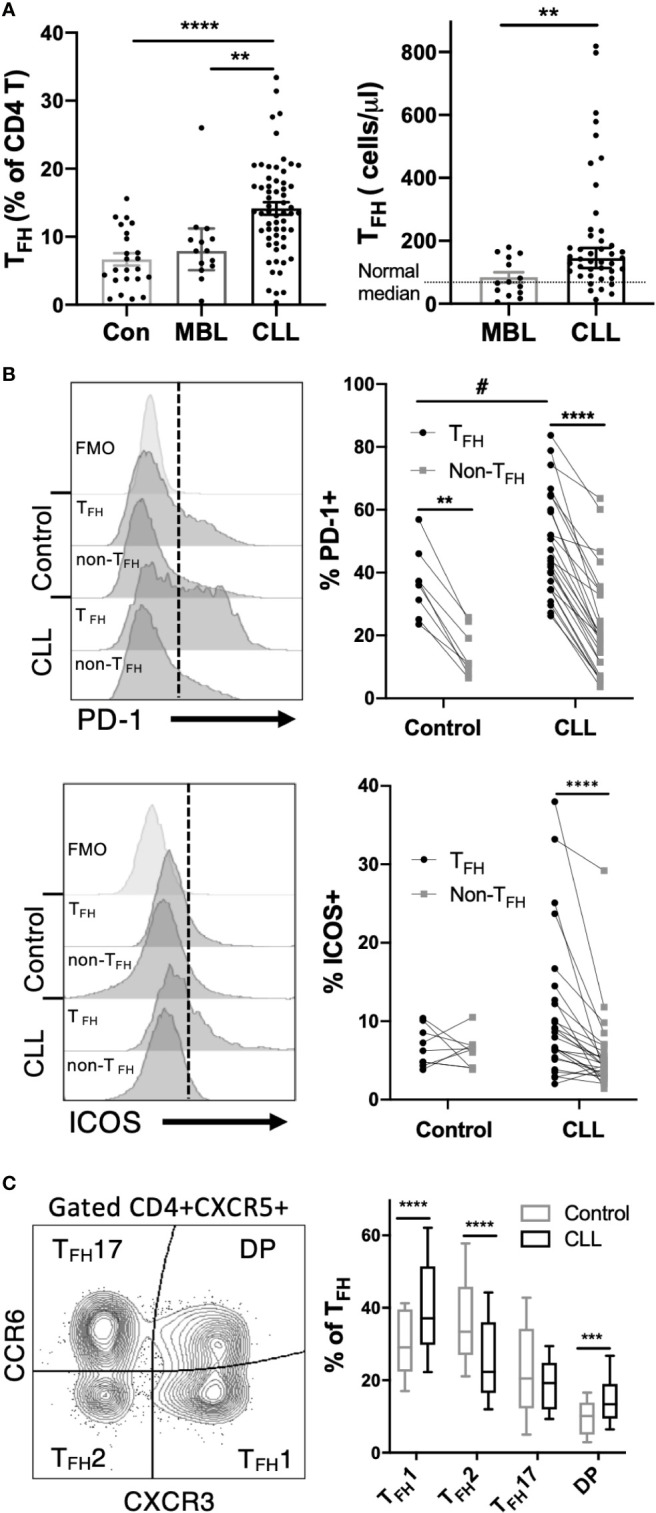
T_FH_ cells are expanded and phenotypically altered in CLL patients. Peripheral blood mononuclear cells (PBMC) were isolated from CLL and MBL patients or control donors and analyzed by flow cytometry. For complete gating strategy see **Supplementary Data**. **(A)** T_FH_ frequency and absolute numbers are significantly increased in CLL patients. N=23 for controls, 14 for MBL and 61 for CLL. All graphs illustrate the individual patient values, median, and 95% confidence interval. Mann-Whitney U test, **(p<0.01), ****(p<0.0001). **(B)** Expression of PD-1 or ICOS on T_FH_ and non-T_FH_ CD4+ T cell populations. T_FH_ were gated as CD4+CXCR5+CD45RA- cells. Individual patients or control values are connected by lines. Histograms labeled FMO show fluorescence minus one staining controls. *denotes significance by Wilcoxon paired t test, ****(p<0.0001). ^#^denotes significance by Mann-Whitney U test, #(p<0.05). **(C)** Proportions of T_FH_1, T_FH_2 and T_FH_17 sub-populations as determined by CCR6 and CXCR3 expression. Mann-Whitney U test, ***(p<0.001), ****(p<0.0001).

Peripheral blood mononuclear cells collected from CLL patients, MBL patients or age-matched controls were analyzed by multicolor flow cytometry to assess T follicular helper (T_FH_) cell populations. Gating on CD4+CXCR5+CD19-CD14- live lymphocytes (**Figure S1**) revealed a significant elevation in both T_FH_ frequencies and overall T_FH_ numbers in CLL but not MBL patients (**Figure 1A**). CLL T_FH_ express higher levels of T_FH_-associated activation markers PD-1 and ICOS than corresponding non-T_FH_ CD4 T cells and more PD-1 than control T_FH_ (**Figure 1B**). Compared to control T_FH_, CLL T_FH_ populations contain a higher proportion of CD45RA-/CCR7- effector memory cells and fewer CD45RA-CCR7+ central memory cells (**Figure S2**). Within the T_FH_ population we further examined T_FH_1, T_FH_2 and T_FH_17 subset composition based on expression of chemokine receptors CXCR3 and CCR6 (24) and found that CLL patients demonstrate significant skewing towards the CXCR3+CCR6- T_FH_1 population (**Figure 1C**). The increased T_FH_1 skewing in CLL patients was accompanied by significantly reduced frequencies of the T_FH_2 population, whereas no significant change in T_FH_17 cells was observed (**Figure 1C**). Together these results indicate that T_FH_ cells are expanded and phenotypically altered in CLL.

### Association of Follicular Helper T Cells With Disease Burden

**Figure 2:**
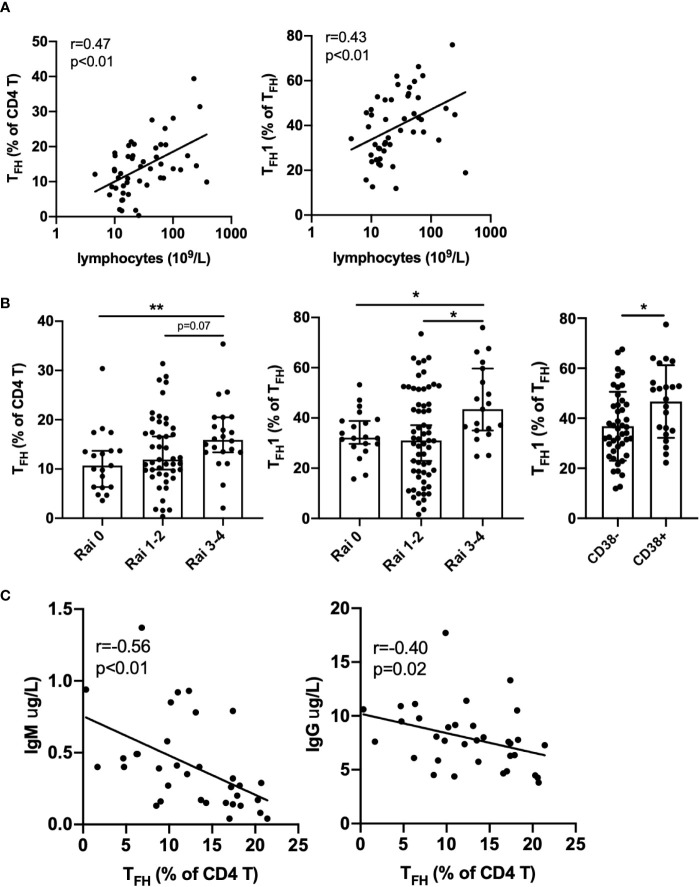
Association of T_FH_ with disease burden and immune deficiency in CLL patients. **(A)** T_FH_ frequency and T_FH_1 skewing are positively correlated with lymphocyte count. **(B)** T_FH_ frequency or T_FH_1 skewing are elevated in advanced Rai stage patients and T_FH_1 frequency is increased in CD38 positive versus CD38 negative patients. All graphs illustrate the individual patient values, median, and 95% confidence interval. **(C)** T_FH_ frequency is negatively correlated with plasma IgM and IgG levels. Significance was determined by Spearman correlation **(A, C)** or Mann-Whitney test, *(p<0.05), **(p<0.01) **(B)**.

Within the spectrum of CLL patients in the cohort analyzed, we examined whether T_FH_ frequencies were associated with clinical parameters or established biomarkers of disease. Strikingly, we found that both frequency of T_FH_ among CD4+ T cells and T_FH_1 skewing were positively correlated with blood lymphocyte counts (**Figure 2A**). T_FH_ expression of PD-1 or ICOS were also positively correlated with lymphocyte count (**Figure S3**). Frequencies of T_FH_ and skewing to T_FH_1 were significantly elevated in high risk CLL (Rai 3-4) relative to low risk CLL (Rai 0) and CD38+ CLL patients showed more T_FH_1 skewing than CD38- patients (**Figure 2B**). The latter is consistent with a report that CD38 expression is driven by the T_H_1 cytokine IFNγ^25^. Interestingly, T_FH_ frequency was inversely correlated with plasma IgM and IgG levels (**Figure 2C**). Together these results suggest that T_FH_ accumulation and selective skewing to a T_FH_1 phenotype occurs in parallel with expansion of the leukemic B cell clone and decline in normal B cell function.

### Impact of Ibrutinib Treatment on T_FH_ Populations

**Figure 3:**
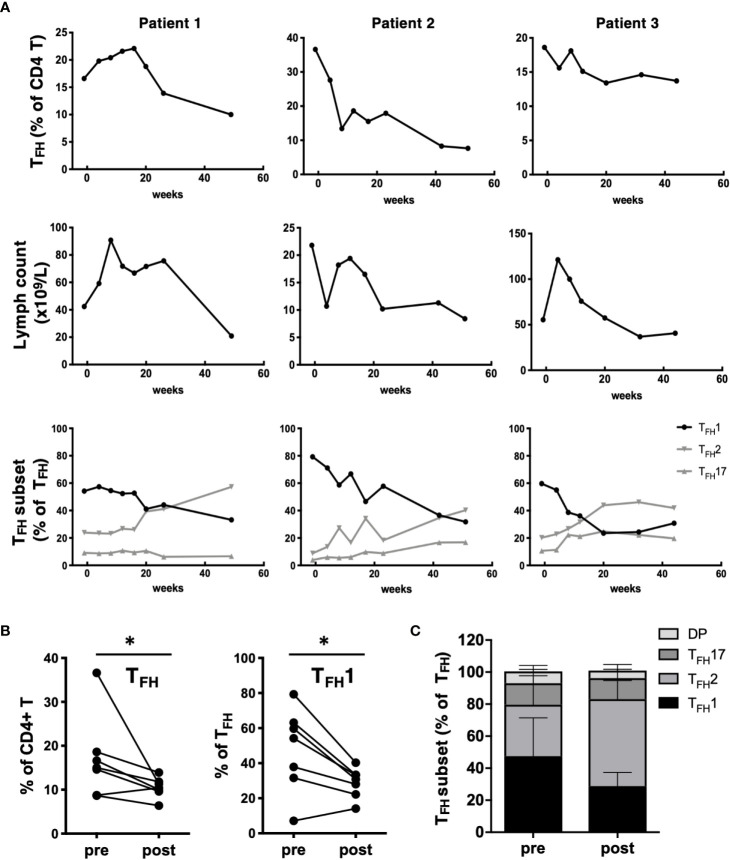
Impact of ibrutinib treatment on T_FH_ populations. **(A)** PBMC samples were collected from CLL patients prior to starting treatment with ibrutinib and at multiple time points over a one-year follow-up. Changes in T_FH_ frequencies, lymphocyte counts or T_FH_ subset frequencies for 3 representative patients over time after ibrutinib treatment for three representative patients are shown. **(B)** Graphs showing pre/post treatment (>40 wk) frequencies of T_FH_ or T_FH_1 subsets, with individual patient data connected by a line (*p<0.05, Wilcoxon test). **(C)** Stacked bar graph summarizing the average composition of four T_FH_ subpopulations pre and post ibrutinib treatment (N=7).

To determine how CLL treatment impacts T_FH_ populations, we examined patients during the first year of ibrutinib treatment. Ibrutinib treatment led to a gradual decline in the frequency of T_FH_ cells over time, in parallel with a decline in total lymphocyte count (**Figure 3A**). In one patient who exhibited prolonged lymphocytosis after treatment, there was a concurrent transient increase in the T_FH_ population prior to normalization. Notably, T_FH_ composition changed post-ibrutinib treatment, with patients exhibiting a gradual re-balancing of T_FH_1, T_FH_2, and T_FH_17 subsets (**Figure 3A**). Significant reductions in both T_FH_ frequencies and T_FH_1 skewing were observed after 40 weeks of treatment (**Figure 3B**). This was accompanied by increased frequencies of CXCR3-CCR6- T_FH_2-like cells, while T_FH_17 frequencies were not altered (**Figure 3C**). These results suggest that ibrutinib treatment can normalize T_FH_ subsets concomitant with reduction in disease burden.

### CLL T_FH_ Cells Produce High Levels of CD40L, TIGIT, IFNγ and IL-21

**Figure 4:**
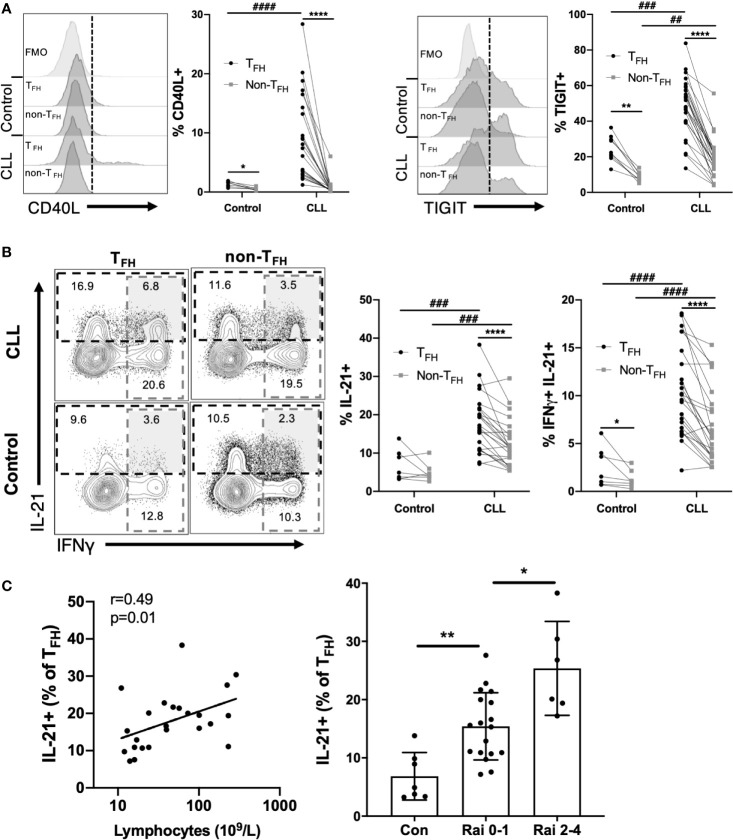
CLL T_FH_ cells express abnormally high levels of CD40L, TIGIT, IFNγ and IL-21. **(A)** Expression of CD40L or TIGIT within T_FH_ and non-T_FH_ CD4+ T cell populations from controls and CLL patients. Left panels show representative staining histograms within the indicated cell populations. Histograms labeled FMO show fluorescence minus one staining controls. Right graphs show percent CD40L or TIGIT positivity among T_FH_ or non-T_FH_ populations, with individual subjects connected by lines. **(B)** Intracellular staining of IFNγ and IL-21 in T_FH_ or non-T_FH_ cell populations. Representative flow plots illustrate gating (left panels). Gated IL-21+ or IFNγ+/IL-21+ cell frequencies within the indicated groups (right panels). In **(A, B)** *denotes significance by Wilcoxon paired t test, ****(p<0.0001); ^#^denotes significance by Mann-Whitney U test, ^##^(p<0.01), ^###^(p<0.001), ^####^(p<0.0001). **(C)** T_FH_ cell expression of IL-21 is associated with lymphocyte count and Rai stage. Left panel, Spearman correlation; Right panel, Mann-Whitney U test, *(p<0.05), **(p<0.01).

We further examined expression of costimulatory molecules and cytokines by CLL T_FH_ cells. We found that T_FH_ express higher levels of CD40L than non-T_FH_ in both CLL patients and controls, however CLL T_FH_ exhibit a strikingly elevated expression of CD40L (approximately 5-fold on average) relative to control T_FH_ (**Figure 4A**). In addition, we found that CLL T_FH_ express high levels of TIGIT (**Figure 4A**), an inhibitory immunoreceptor previously thought to function in T:B cell interactions (25, 26). We examined the ability of CLL T_FH_ to produce the canonical Type 1 cytokine IFNγ and the T_FH_-associated cytokine IL-21 (**Figure 4B**). Remarkably, we observed a substantial increase in both the frequency IL-21 producing and IL-21/IFNγ double-producing cells in CLL patients, with the CLL T_FH_ population containing significantly more IL-21 and double-producing cells than control T_FH_ cells (**Figure 4B**). T_FH_1 cells produced significantly more IL-21 and IFNγ than other T_FH_ subsets, but interestingly produced slightly less CD40L (**Figure S4**). We further assessed whether levels of CD40L, TIGIT or IL-21 expression by T_FH_ are associated with disease burden or stage. Interestingly, while CD40L and TIGIT expression did not show strong associations, IL-21 expression by T_FH_ was significantly associated with lymphocyte count and Rai stage (**Figure 4C**). These results indicate that CLL T_FH_ cells produce abnormally high levels of costimulatory molecules and cytokines known to stimulate CLL survival and proliferation and the expression of IL-21 by these cells is associated with adverse biomarkers and disease burden.

### Activated T_FH_1-Like Cells Are Present in CLL Lymphoid Tissues

**Figure 5:**
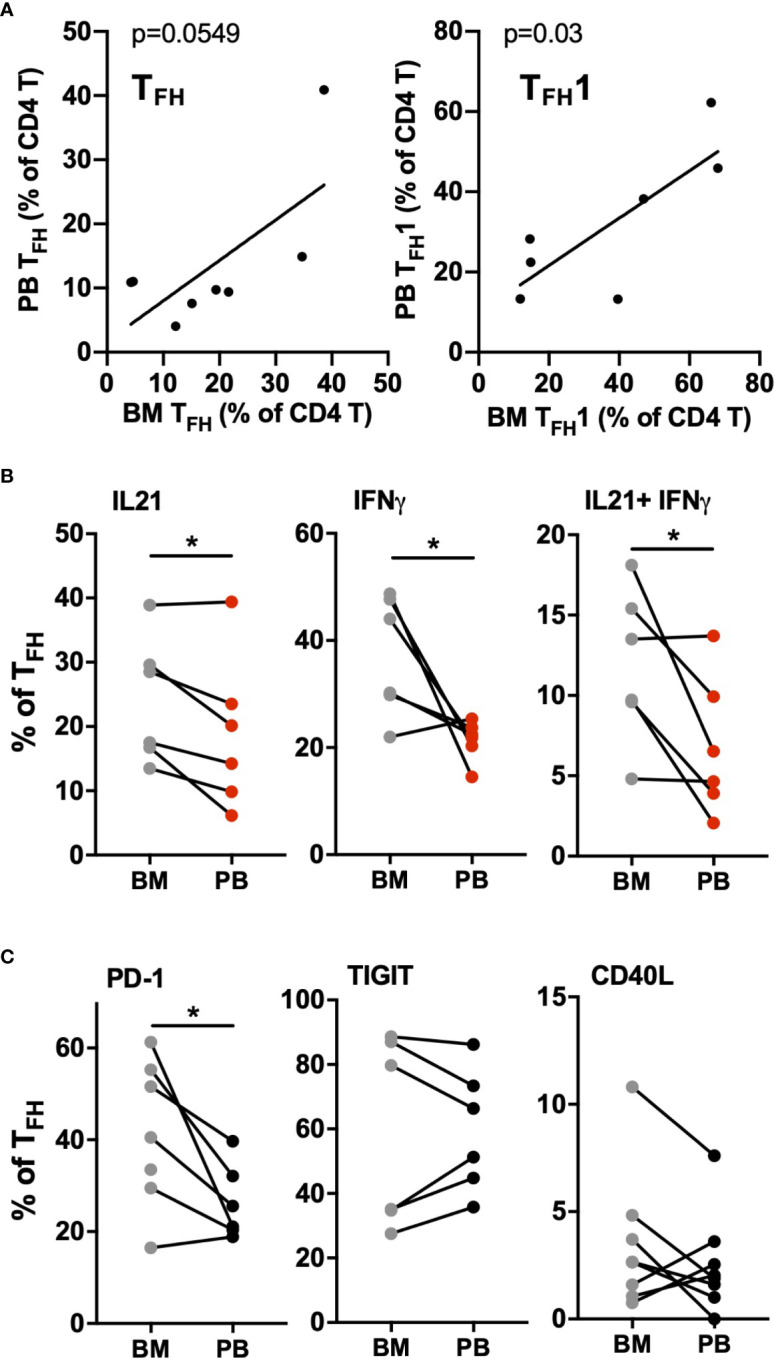
CLL bone marrow contains high levels of activated T_FH_1 cells. Mononuclear cells isolated from bone marrow aspirates (BM) and peripheral blood samples (PB) of the same patients were analyzed by flow cytometry. **(A)** Correlation between T_FH_ (left) or T_FH_1 (right) frequencies in marrow versus matched blood samples **(B)** Comparison of cytokine production in marrow versus blood T_FH_ populations. **(C)** Comparison of activation/costimulatory markers in marrow versus blood T_FH_ populations. * denotes significance by Wilcoxon paired t test, *(p<0.05).

**Figure 6:**
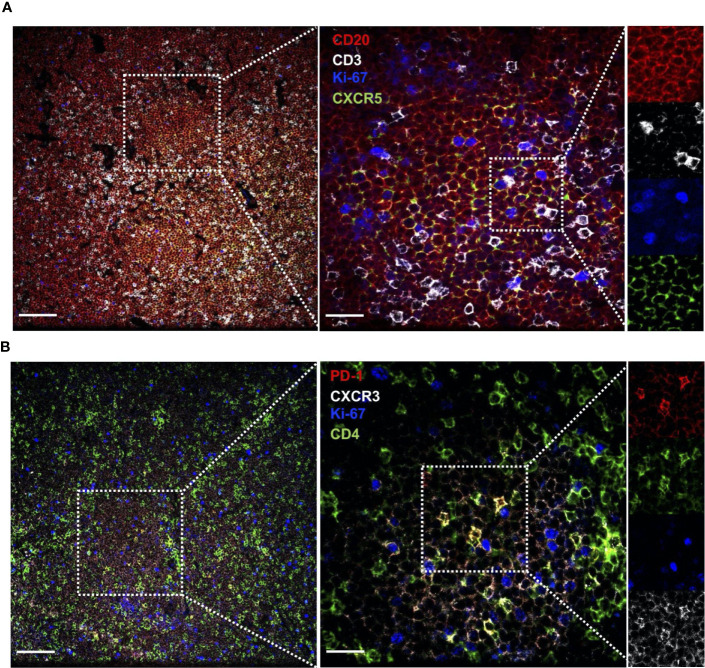
CLL lymph nodes contains cells expressing activated T_FH_1 markers. Lymph node sections from CLL patients were stained with the indicated labelled antibodies and imaged by confocal microscopy. **(A)** Section stained for CLL B cell marker CD20, T cell marker CD3, proliferation marker Ki67 and CXCR5 (expressed on CLL B cells and T_FH_). **(B)** Section stained for activation marker PD-1, T cell marker CD4, proliferation marker Ki67 and CXCR3 (expressed at low levels on CLL B cells and high levels on T_FH_1 cells). Several magnifications are shown (Left panel scale bar = 100µm, right panel scale bar = 25µm) to illustrate T cell presence within CLL B cell clusters containing proliferating cells, and close contact between CLL cells and T cells expressing CD3, CD4, CXCR5, PD-1 and CXCR3.

CLL cells are present at varying levels in the bone marrow and lymph nodes, and signals present in these microenvironments are thought to drive CLL proliferation. To investigate whether circulating T_FH_ cells may represent the counterpart of T_FH_ populations present within lymphoid tissues, paired blood and bone marrow samples from CLL patients were analyzed. We found a significant correlation between blood and marrow T_FH_ and T_FH_1 cell frequencies from the same patients (**Figure 5A**). Interestingly, T_FH_ populations in bone marrow showed increased activation status relative to those in peripheral blood of the same patients, expressing significantly more IL-21, IFNγ and IL-21/IFNγ double-producing cells (**Figure 5B**). While levels of PD-1 was also elevated, CD40L and TIGIT were similar in bone marrow and peripheral blood T_FH_ (**Figure 5C**). We further examined lymph node tissue sections from CLL patients using immunofluorescent staining and could identify T cells present within proliferation centers expressing the T_FH_1 markers CD3, CD4, CXCR5, CXCR3 and PD-1 (**Figure 6**). These results suggest that T_FH_ cells are present in both circulation and within lymphoid tissues, with the latter showing similar skewing to T_FH_1 and a highly activated phenotype.

### Autologous Activated CD4 T Cells Can Promote CLL B Cell Survival, Activation and Proliferation

**Figure 7:**
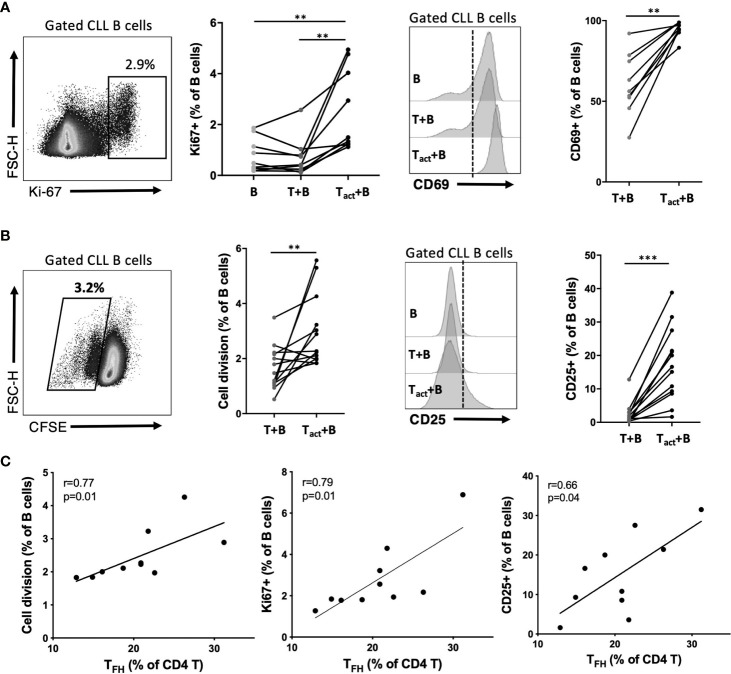
Impact of activated autologous CD4+ T cells on CLL activation and proliferation. Purified CLL cells were co-cultured with purified autologous CD4+ T cells from the same PBMC sample. Prior to co-culture, CLL cells were labeled with CFSE and T cells were briefly exposed to an activation cocktail (T_act_) or kept in medium alone (T). After 2 or 6 days of co-culture, cells were analyzed by flow cytometry to determine cell division and expression of activation markers. B cells were gated as live CD19+CD4- cells. **(A)** Expression of nuclear proliferation antigen Ki67 or activation marker CD69 at day 2 of culture. **(B)** CLL cell division assessment based on CFSE dilution and expression of activation marker CD25 at day 6 of culture. In **(A, B)** *denotes significance by Wilcoxon paired t test, **(p<0.01), ***(p<0.001). **(C)** Correlation of observed B cell division, Ki67 expression or CD25 expression with the frequency of T_FH_ within CD4 T cells for each patient sample cultured (Spearman correlation).

We developed an *in vitro* system to study CLL B cell interaction with autologous CD4+ T cells. CD4 T cells were isolated from patient PBMC, pre-cultured overnight with a T cell-activation cocktail or medium alone, then washed and co-cultured with B cells isolated from the same patient that were labeled with cell division tracking dye CFSE. Resulting CLL-B cell division, and expression of activation markers by B and T cells, were assessed after 2 and 6 days of co-culture. At day 2, a significant increase in CLL B cell expression of CD69 and nuclear proliferation antigen Ki-67 was observed in the presence of activated CD4 T cells, compared to co-cultures with non-activated T cells or B cells alone (**Figure 7A**). Activated CD4 T:B cell co-culture also promoted an increase in CD25 activation marker expression among B cells and increased CLL B cell survival at this time point, whereas increased CD38 expression or cell division (as assessed by CFSE dilution) were not observed at day 2 (**Figure S5A**). After 6 days of co-culture, CLL-B cell division was observed (**Figure 7B**) and most divided cells also expressed Ki-67 (**Figure S5B**). At day 6, CLL B cells also exhibited significant upregulation of the activation marker CD25 (**Figure 7B**) as well as CD38 and CD69 (**Figure S5C**) when co-cultured with activated CD4 T cells. Notably, there was a positive correlation between patient T_FH_ frequency and the frequency of divided, Ki-67+ and CD25+ CLL B cells observed in co-cultures (**Figure 7C**), consistent with a potential role for T_FH_ cells in driving CLL-B cell activation and proliferation.

### CLL Cells Activate T_FH_ Cells in Co-Culture

**Figure 8:**
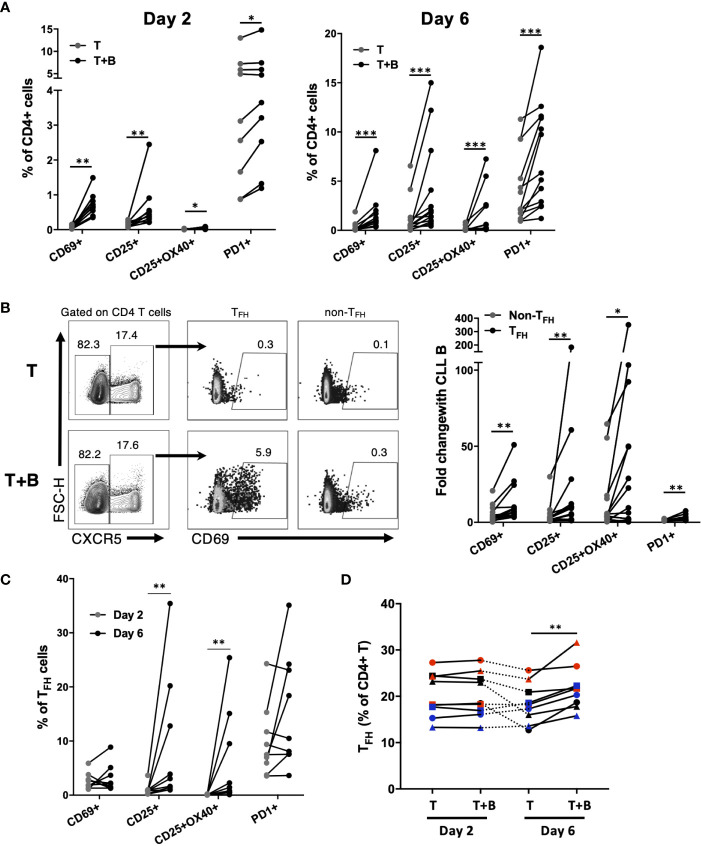
Impact of CLL B cells on activation of autologous CD4+ T cell subsets. Purified CLL cells were co-cultured with purified autologous CD4+ T cells that were not pre-activated. **(A)** Expression of activation markers on CD4+ T cells after 2 days or 6 days of co-culture. **(B)** Example flow cytometry analysis showing expression of CD69 on T_FH_ or non-T_FH_ CD4+ T cells. Data in right panel is expressed as fold change in activation marker expression among T_FH_ or non-T_FH_ (calculated as percent expression in T+B cell co-culture/percent expression in T cell only culture). **(C)** CD25 and OX40 expression increase on T_FH_ cells co-cultured with CLL B cells between day 2 and day 6. **(D)** Patient-specific frequencies of T_FH_ are maintained in CLL B cell co-culture. Individual patients are connected by lines. *denotes significance by Wilcoxon paired t test, *(p<0.05), **(p<0.01), ***(p<0.001).

During CLL:T cell interactions, CLL-B cells can also impact CD4+ T cell activation (27), but it is unclear whether particular cell subsets are preferentially targeted. In order to assess CLL B cell-dependent activation of CD4+ T cell subsets in co-culture, CD4 T cells untreated with activation cocktail were assessed for their expression of various activation markers in the presence or absence of autologous CLL cells. It was found that the presence of CLL B cells led to an increase in expression of activation markers in a small fraction (less than 15%) of autologous CD4 T cells (**Figure 8A**). This upregulation was significant for CD25, CD69 and PD-1 within 2 days of co-culture (**Figure 8A**). The frequency of CD4 T cells co-expressing both CD25 and OX40, associated with antigen-specific T cell recall responses (28), was also increased in the presence of the B cells at both timepoints (**Figure 8A**). To determine whether T_FH_ cells were preferentially activated by CLL-B cells, we assessed activation marker expression on T_FH_ and non-T_FH_ fractions. The presence of B cells provoked an increase in CD69+ cells in both populations, however the fold increase in expression was significantly higher for T_FH_ (**Figure 8B**). In addition to CD69, T_FH_ cells showed greater fold increases in expression of CD25, CD25/OX40 and PD-1 than non-T_FH_ cells when co-cultured with CLL (**Figure 8B**). T_FH_ cell expression of activation markers CD25 and OX40 increased between day 2 and day 6 of culture, while no further increase in CD69 or PD-1 were observed after day 2 (**Figure 8C**). We noted that while the patient-specific frequencies of T_FH_ cells were very stable over the first two days of culture, they began to decline by day 6 in cultures without B cells but were well maintained in the presence of CLL B cells (**Figure 8D**). Taken together, these results indicate that in this co-culture system, CLL-B cells can promote T_FH_ cell activation and maintenance.

## Discussion

Abnormalities in T cell subsets and function in CLL have been reported in a number of studies, including changes in CD4/CD8 ratios, expansion of the Treg population, loss of naïve and increased memory populations, and increased expression of exhaustion markers (23, 29, 30). Here we report an in-depth analysis of follicular helper T cell subsets and functional status as well as their association with disease progression, immune status and ibrutinib treatment. Our results are partially consistent with studies from Asian and UK patients which also observed elevated T_FH_ frequencies in CLL (31, 32). Our study reveals that elevation in T_FH_ cells is first observable in Rai 0 CLL patients, whereas no increase was apparent in the MBL group. These previous studies reported lack of association with IgVH mutation status (31, 32) and we also found no association with either IgVH mutation status or ZAP-70 status (data not shown). Our study demonstrates a significant positive correlation between T_FH_ frequencies and total lymphocyte counts, indicating that increasing acquisition of the T_FH_ phenotype among CD4+ T cells occurs in tandem with expansion of the malignant B cell clone. A previous study found increased T_FH_ frequencies in Binet C versus A/B patients (32), consistent with our observed trends of higher T_FH_ frequencies, in the Rai 3-4 group. Surprisingly, we further find that expanded T_FH_ populations in advanced stage CLL exhibit significant T_FH_1 skewing. Strikingly, increased expression of PD-1, ICOS and IL-21 by T_FH_ cells were associated with disease burden, and CLL T_FH_ cells were also found to over-express other stimulatory molecules such as IFNγ, TIGIT and CD40L.

Together our findings indicate that CLL T_FH_ express more PD-1 than either non-T_FH_ CD4+ T cells from CLL patients or T_FH_ from controls. PD-1 is a marker for chronically-activated or exhausted T cells, and was previously reported to be elevated in CLL CD4+ T cells (33). Our results are consistent with this finding, and further define the CD4 sub-populations abnormally expressing this marker. In the context of the T_FH_ literature, the PD-1+ T_FH_ phenotype which we describe here (PD-1+CXCR5+CCR7low) is not associated with functional exhaustion but rather has been previously associated with recent T_FH_ activation in tissues and enhanced functional capacity for providing B cell help (34, 35). However, we found evidence that T_FH_ frequencies are *negatively* correlated with IgM and IgG levels. This finding is consistent with these T_FH_ cells being dysfunctional in relation to their normal supportive roles for antibody responses; or alternatively it may reflect the association of T_FH_ expansion with expansion of the CLL clone, and concomitant disruption of normal B cell niches within tissues.

Our results indicate that ibrutinib can normalize T_FH_ frequencies and activation gradually over several months of treatment. These results are partially consistent with previous studies which found that ibrutinib can reduce T cell expression of PD-1 as well as memory markers (36, 37). The effects of ibrutinib on T cells may be due to inhibition of the kinase ITK expressed in T cells, since treatment with acalabrutib, which does not inhibit ITK, showed lesser impact on T cells (37). Patients with BTK mutations were found to have severe deficiency in T_FH_ cells (38), suggesting the possibility that BTK itself may be required for T_FH_ development and/or maintenance. Alternatively, ibrutinib may indirectly affect T_FH_ cell populations *via* depletion of the CLL B cell clone and/or its other effects on the immune microenvironment.

Previous studies have provided considerable evidence that blood T_FH_ are clonally related to T_FH_ in lymphoid tissues. We found cells expressing markers of activated T_FH_ cells within proliferation centers in lymph nodes and in bone marrow, suggesting they are in direct contact with CLL B cells within these microenvironments. T_FH_ cells present in the bone marrow expressed a more activated phenotype than peripheral blood T_FH_, including highly elevated PD-1 and IL-21/IFNγ expression in some patients, indicating that lymphoid tissues are likely their primary site of activation. Given that IFNγ can impair hematopoietic stem cell function and contribute to bone marrow failure (39), it is possible that the presence of these activated T cells in marrow may directly participate in disruption of marrow function in advanced stage disease.

CLL T_FH_ cells exhibit elevated expression of B cell stimulatory molecules CD40L and IL-21. Numerous studies have shown that the culture with CD40L-expressing T or stromal cell lines promotes CLL-B cell proliferation (40–42). The addition of IL-21 to CD40L-stimulated CLL-B cells increases the frequency of divided cells and the average number of divisions (31, 42). Our study provides evidence that T_FH_ cells are the predominant T cell subset producing these CLL stimulatory factors *in vivo*. Additionally, we observed that CLL T_FH_ cells expressed high levels of TIGIT, molecule that has been linked to CLL-B cell survival (43). T_FH_1 cells are reported to be relatively poor helpers for normal B cell responses (24), however it is possible that the abnormal T_FH_1-like cells in CLL are adapted to support malignant CLL B cells. A study using a mouse xenograft model found that T cells driving CLL cell proliferation had a T_H_1 phenotype (44), indicating the potential of these cells to serve as supportive cells in the microenvironment. Some studies have implicated IFNγ as a supportive cytokine for human CLL cells (27, 45). Together, our data demonstrate that abnormal T_FH_ present in CLL patients over-produce multiple factors that could potentially support CLL survival and proliferation in tissues.

Our *in vitro* co-culture studies revealed evidence that CLL cells can preferentially activate T_FH_ and that T_FH_ expansion is associated with ability of activated CD4+ T cells to trigger CLL proliferation *in vitro*. Activated autologous CD4 T cells promoted CLL cell activation marker expression and division in line with other studies showing that the expression of activation markers by CLL cells is increased after 2-3 days of co-culture with T cells (27, 45), while CLL division is only observed after 4 days (27, 40, 46). To our knowledge, this is the first study to report this functional activity of purified autologous CD4 T cells in CLL, without addition of other factors such as stromal cells or cytokines. We observe that reactivation of CD4+ T cells using anti-CD3/28 is required to observe affects on CLL B cells, which may reflect that blood T_FH_, like blood CLL cells, are relatively quiescent compared to their counterparts in lymphoid tissues. Notably, the frequency of T_FH_ cells within the CD4 T cell pool positively correlated with CLL-B cell division; however we were unable to purify sufficient numbers of T_FH_ from CLL patient blood to carry out co-culture studies. Thus, while our data are consistent with a role of T_FH_ cells in driving CLL proliferation, it remains possible that increased frequency of T_FH_ could be associated with other alterations in CD4 T cell or autologous CLL cell populations that are critical for proliferation under these conditions.

Notably, the reciprocal activation of T_FH_ cells by CLL cells in co-culture included induction of CD25/OX40 double positive T_FH_ cells. Co-expression of these activation markers has been shown to be dependent on antigen-specific stimulation (28). Previous studies have raised the possibility of antigen-specific cognate interactions between CLL and CD4+ T cells leading to oligoclonal expansions (47), however the phenotype of these oligoclonal T cells has not been determined. One study found that CLL : CD4 T cell interactions *in vitro* can be abrogated by an anti-pan-MHC II antibody (27), consistent with cognate interaction. In addition to induction of activation marker expression on T_FH_, we found that CLL cells could maintain T_FH_ frequencies during *in vitro* culture, resulting in significantly higher T_FH_ frequencies in the presence of CLL after 6 days. These results suggest that direct bi-directional interactions between CLL cells and abnormal T_FH_ cells is a significant feature of the dysfunctional CLL immune microenvironment.

Together, our findings suggest that alterations in T_FH_ frequencies, activation status, subset distribution and costimulatory molecule expression can serve as alternative biomarkers in CLL reflective of lymphoid tissue involvement and disease progression. T_FH_ cells may represent significant sources of CLL stimulatory molecules and play roles in disrupting normal immune function and promoting CLL proliferation.

## Data Availability Statement

The raw data supporting the conclusions of this article will be made available by the authors, without undue reservation.

## Ethics Statement

The studies involving human participants were reviewed and approved by Research Ethics Board at the University of Manitoba. The patients/participants provided their written informed consent to participate in this study.

## Author Contributions

XW and JF performed research, analyzed data, performed statistical analyses and wrote the manuscript. CZ performed research and analyzed data. SG, VN, VB and JJ designed research and collected CLL patient samples. AM designed research, analyzed and interpreted data, supervised trainees and wrote the manuscript. All authors contributed to the article and approved the submitted version.

## Funding

XW and JF received graduate studentship funding from Research Manitoba. This study was supported by the Manitoba Tumour Bank, a member of the Canadian Tissue Repository Network, funded in part by the CancerCare Manitoba Foundation and the Canadian Institutes of Health Research. This study was supported by grants from the Leukemia and Lymphoma Society of Canada and Research Manitoba.

## Conflict of Interest

VB and JJ: Consultant and research funding from Astra Zeneca, Abbvie, Janssen and Gilead unrelated to this work. VB: Received fees from BIOGEN for compounds unrelated to this work. SG: Research funding from Gilead, Abbvie and Janssen unrelated to this work.

The remaining authors declare that the research was conducted in the absence of any commercial or financial relationships that could be construed as a potential conflict of interest.
