# Supplementary material for: Altered T Follicular Helper Cell Subsets and Function in Chronic Lymphocytic Leukemia
Source: Front Oncol. 2021 Apr 28;11:674492. doi: 10.3389/fonc.2021.674492 (PMC8113764; doi:10.3389/fonc.2021.674492)
Supplement: Supplementary file 1 [file DataSheet_1.pdf]

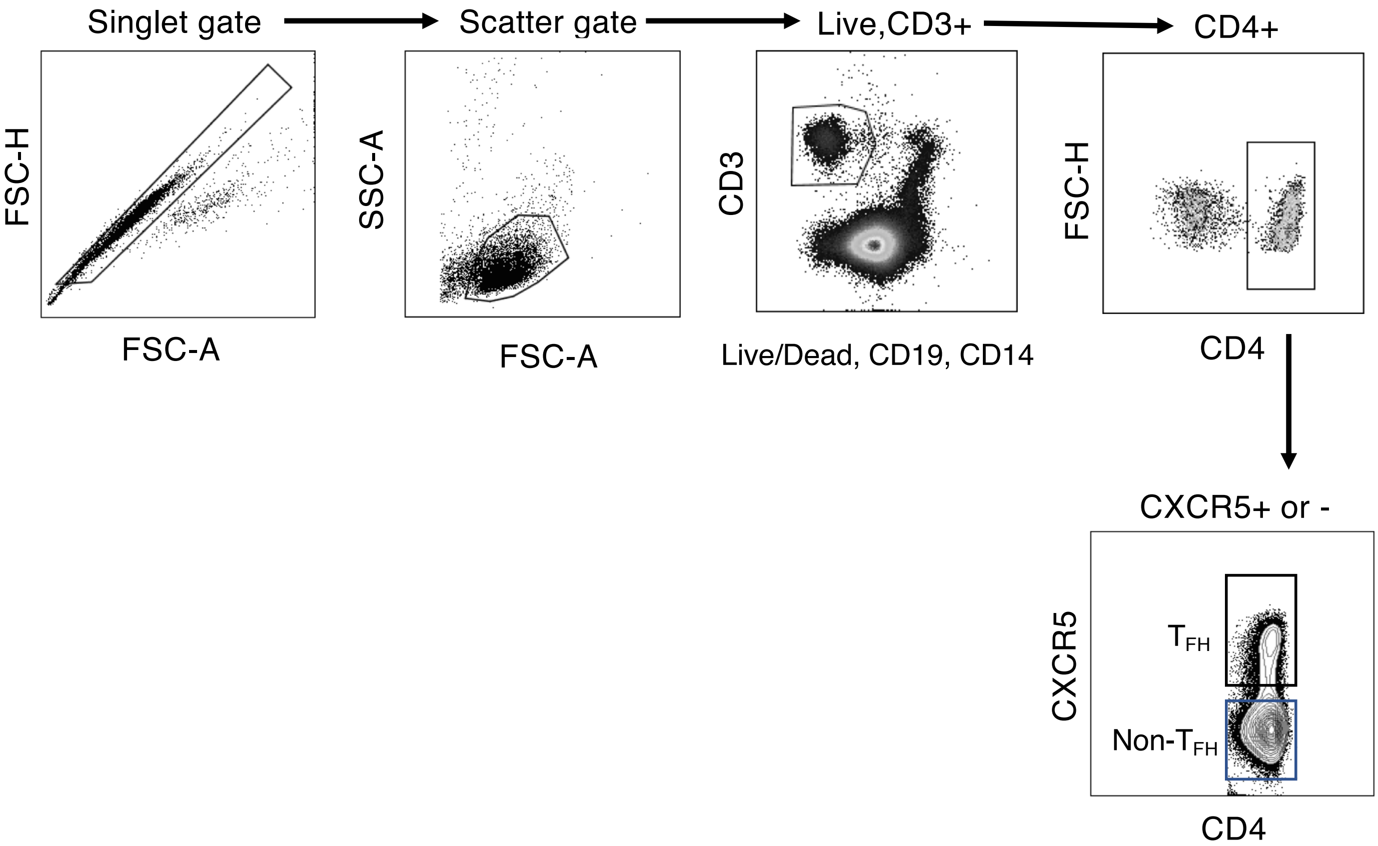

Figure S1. Flow cytometry gating strategy

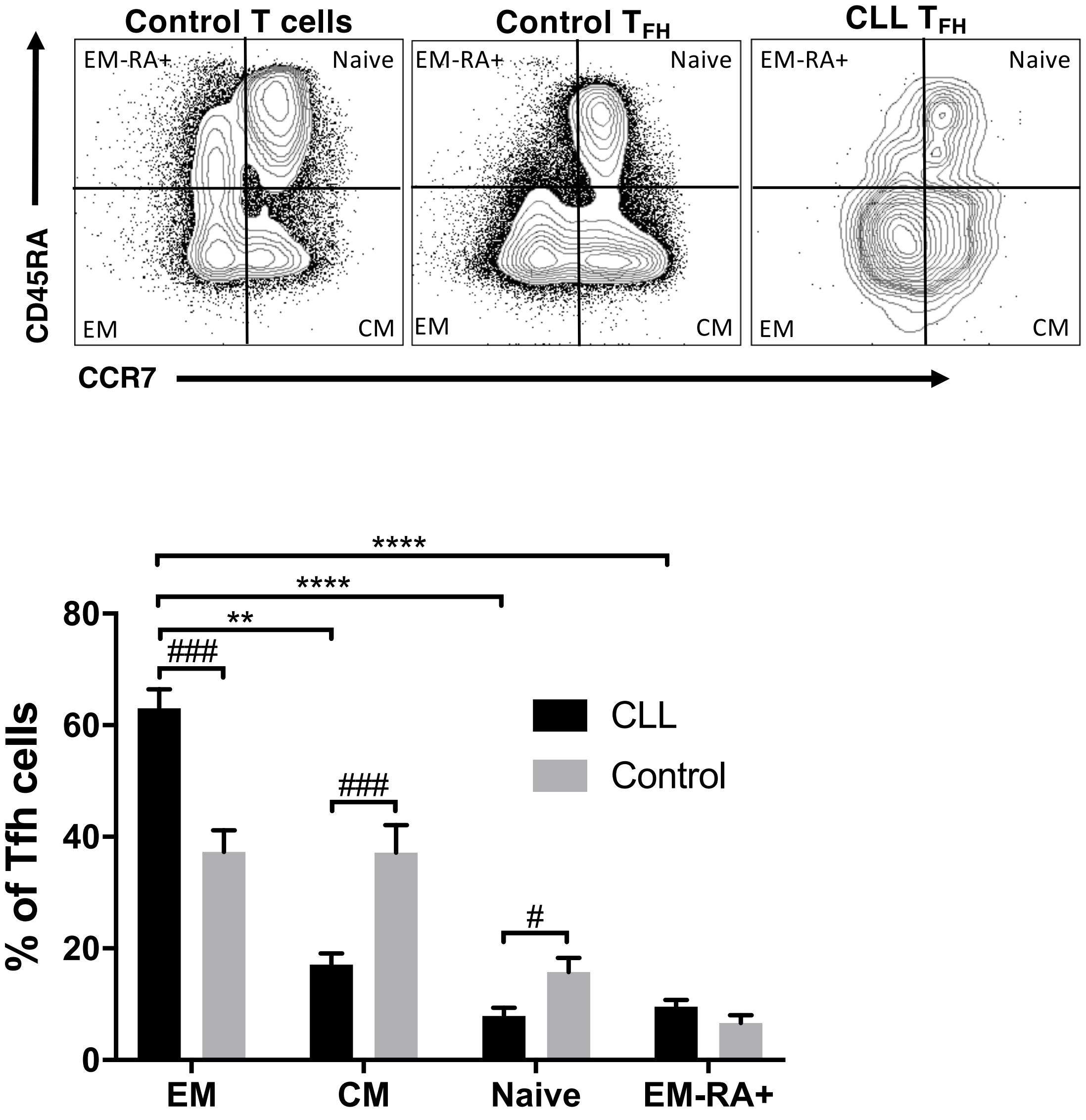

Figure S2. CLL T_{FH} have predominantly an effector memory phenotype
CD4+ T cells gated as CXCR5+ T_{FH} or CXCR5- non-T_{FH} populations were then subdivided based on CCR7 and CD45RA expression into naïve, effector memory (EM), central memory (CM), or EM CD45RA+. Top panels show example gating. Bottom panels show summary data for 19 CLL and 7 controls. Notably, CLL T_{FH} were significantly enriched in effector memory phenotype cells compared to control T_{FH}.

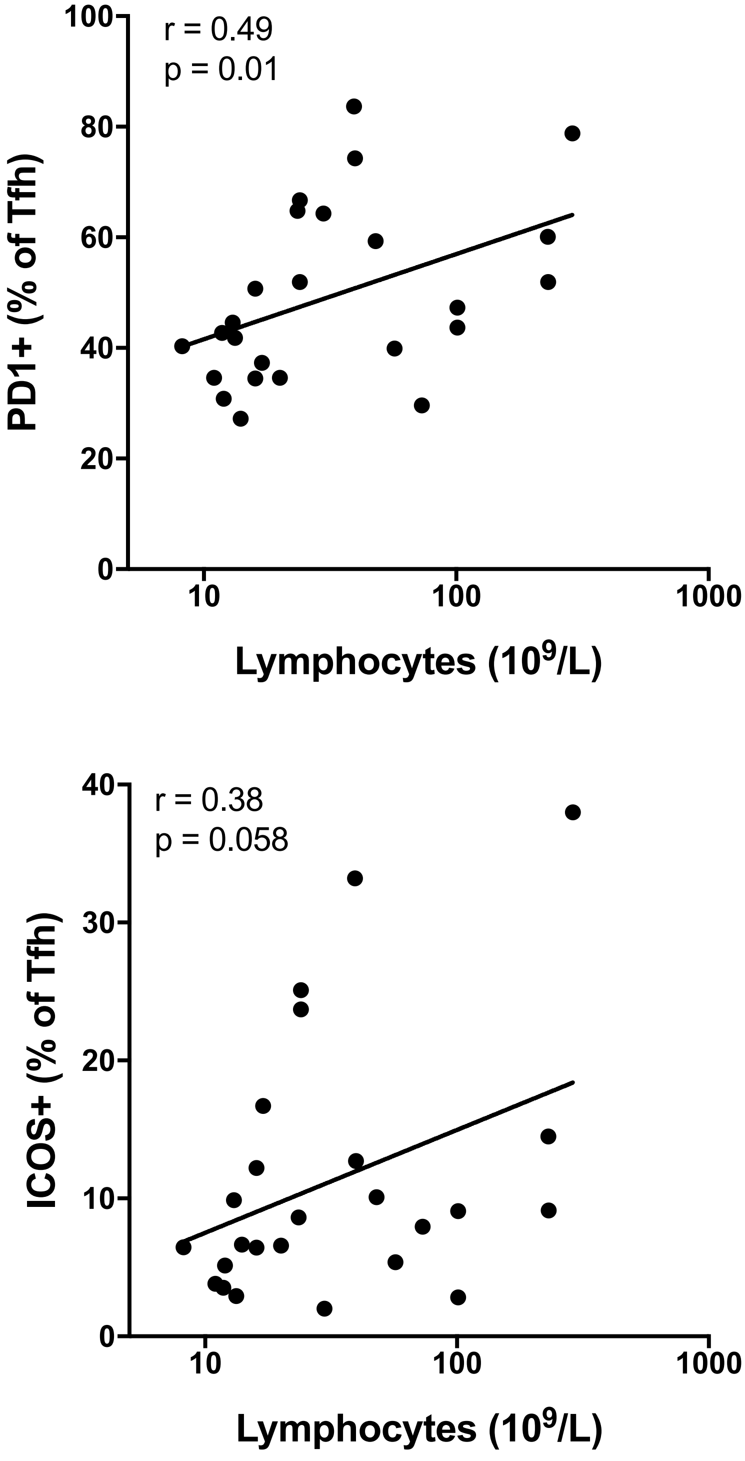

Figure S3. CLL T_{FH} expression of PD-1 and ICOS correlates with patient lymphocyte count. T_{FH} were gated as CD4+CXCR5+CD45RA- and percent PD-1+ or ICOS+ cells were determined for each patient.

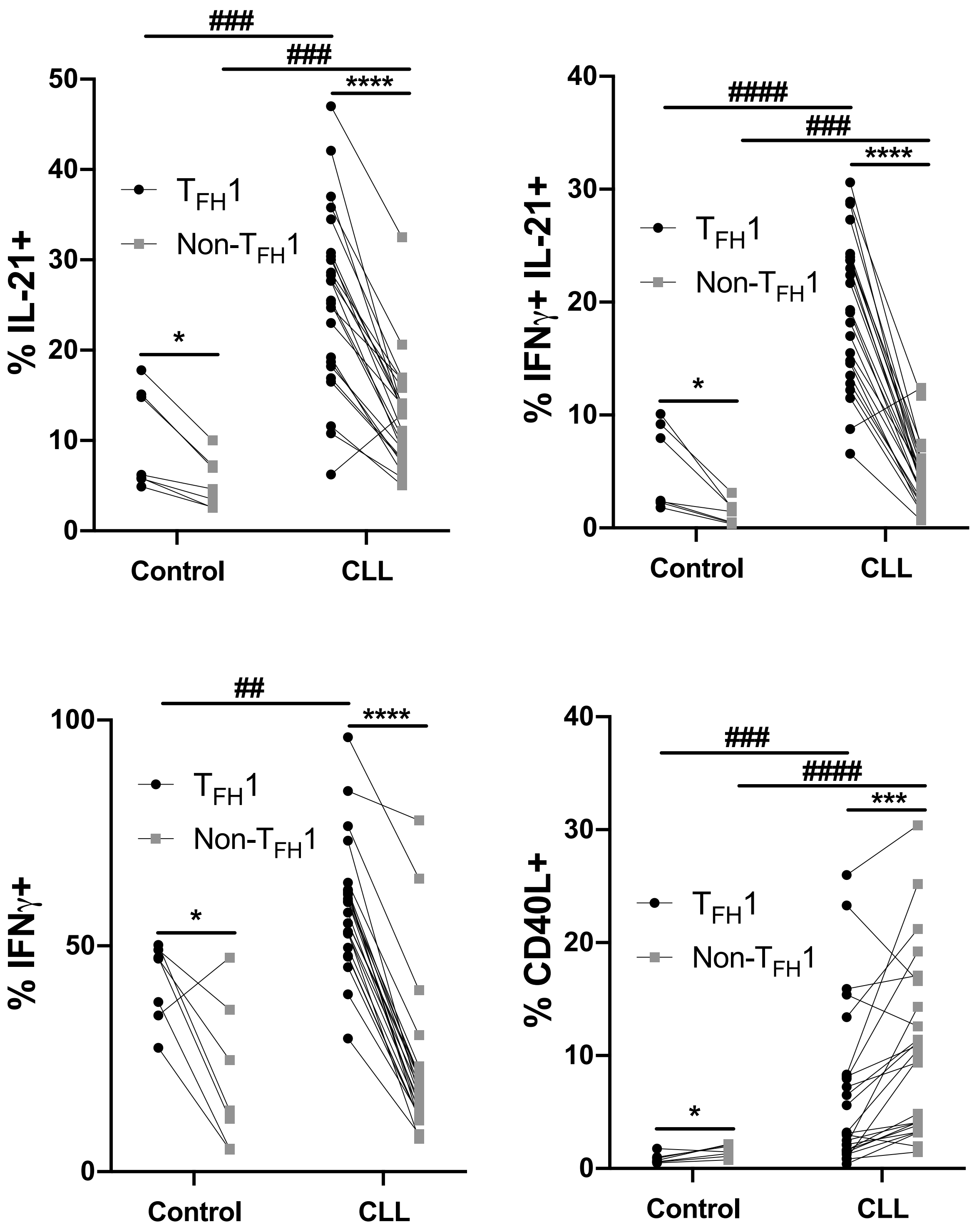

Figure S4. CLL $T_{FH}1$ cells produce more IL-21 and IFNγ but less CD40L than other T_{FH} subsets.

CXCR3+CCR6- $T_{FH}1$ cells were compared for expression of IL-21, IFNγ and CD40L relative to all remaining non-$T_{FH}1$ cells within the CD4+CXCR5+ gate.

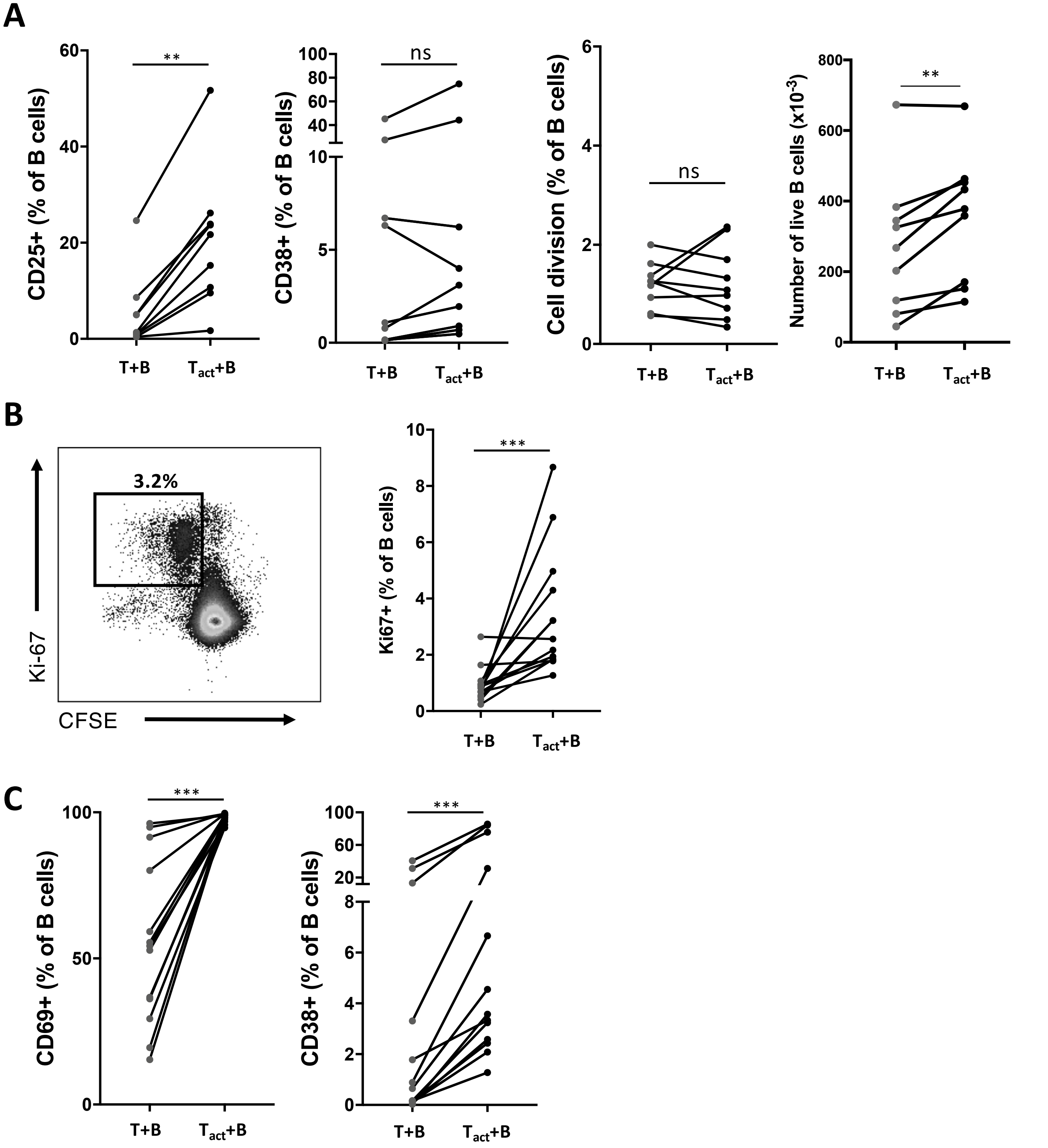

Figure S5. Impact of activated CD4+ T cells on CLL activation and proliferation. CLL cells were co-cultured with autologous CD4+ T cells which had been cultured with activation cocktail (T_{act}) or medium alone (T). **(A)** After 2 days of co-culture, CLL cell expression of CD25, CD38, cell division (assessed by CFSE dilution) or number of live cells were determined. Individual patient results are connected by lines. **(B)** Expression of of Ki-67 on CFSE-low divided B cells after 6 days of co-culture. **(C)** Expression of activation markers CD69 and CD38 on B cells after 6 days of co-culture.

Target	Fluorochrome	Company	Catalog No.	Recommended µL/sample*	Titrated µL/sample*	Surface or Post-fix/perm
**Cell surface marker panel**						
CCR6	BV421	BD Biosciences	562515	5	2.5	surface
CD19	V500	BD Biosciences	561121	5	1.25	surface
CD14	V500	BD Biosciences	561391	5	1.25	surface
CD45RA	BV605	BD Biosciences	562886	5	0.625	surface
ICOS	BV650	BD Biosciences	563832	5	2.5	surface
CCR7	BB515	BD Biosciences	565869	5	2.5	surface
CD4	PerCP-Cy5.5	BD Biosciences	341654	20	2.5	surface
CXCR5	PE	eBioscience	12-9185-42	5	1.25	surface
CXCR3	PE-CF594	BD Biosciences	562451	5	1.25	surface
TIGIT	PE-Cy7	BioLegend	372714	10	1.25	surface
PD-1	APC	BD Biosciences	558694	20	5	surface
CD3	APC-H7	BD Biosciences	560176	5	1.25	surface
*Amount to stain 1x10^6 cells in 100 uL						
**Intracelular marker panel**						
CCR6	BV421	BD Biosciences	562515	5	2.5	surface
CD19	V500	BD Biosciences	561125	5	2.5	surface
CD14	V500	BD Biosciences	561392	5	2.5	surface
CD40L	BV605	BioLegend	310826	5	2.5	post-fix/perm
IFN gamma	BV786	BD Biosciences	563731	5	2.5	post-fix/perm
CD4	PerCP-Cy5.5	BD Biosciences	341654	20	20	post-fix/perm#
CXCR5	PE	eBioscience	12-9185-42	5	1.25	surface
CXCR3	PE-CF594	BD Biosciences	562451	5	2.5	surface
IL-21	APC	BioLegend	513008	5	5	post-fix/perm
CD3	APC-H7	BD Biosciences	560176	5	2.5	surface
# stained intracellularly due to reduction of surface CD4 upon PMA/ionomycin stimulation						
**Co-culture analysis panel**						
CCR6	BV421	BD Biosciences	562515	5	2.5	surface
CD14	V500	BD Biosciences	561391	5	2.5	surface
CD4	BV605	BD Biosciences	562658	10	1.25	surface
OX40	BV650	BD Biosciences	563658	10	2.5	surface
Ki-67	BV786	BD Biosciences	563756		5	post-fix/perm
Proliferation	*CFSE*					
CD38	PerCP-Cy5.5	BD Biosciences	551400	10	5	surface
CXCR5	PE	eBioscience	12-9185-42	5	1.25	surface
CXCR3	PE-CF594	BD Biosciences	562451	5	1.25	surface
CD69	PE-Cy7	BD Biosciences	557745	10	2.5	surface
PD-1	APC	BD Biosciences	558694	20	5	surface
CD19	Alexa Fluor 700	BD Biosciences	557921	5	2.5	surface
CD25	APC-H7	BD Biosciences	560225	10	2.5	surface

Table S1. Flow cytometry antibodies

Target	Fluorochrome	Host species	Mono or polyclonal	Company	Catalog No.
CD3	n/a	mouse	monoclonal	abcam	ab17143
CD20	n/a	rabbit	monoclonal	abcam	ab64088
CD4	n/a	rabbit	monoclonal	abcam	ab133616
CXCR3	n/a	mouse	monoclonal	abcam	ab64714
CXCR5	Alexa Fluor 488	rabbit	monoclonal	abcam	ab223980
PD-1	Alexa Fluor 647	mouse	monoclonal	abcam	ab220301
Ki-67	BV421	mouse	monoclonal	BD Biosciences	562899
Mouse IgG1	Alexa Fluor 568	goat	polyclonal	Invitrogen	A-21124
Rabbit IgG	Alexa Fluor 488	goat	polyclonal	Invitrogen	A-11034
Rabbit IgG	Alexa Fluor 647	goat	polyclonal	Invitrogen	A-21246

Table S2. Immunofluorescence microscopy antibodies
